# An Esrrb and Nanog Cell Fate Regulatory Module Controlled by Feed Forward Loop Interactions

**DOI:** 10.3389/fcell.2021.630067

**Published:** 2021-03-19

**Authors:** Ana Sevilla, Dimitri Papatsenko, Amin R. Mazloom, Huilei Xu, Ana Vasileva, Richard D. Unwin, Gary LeRoy, Edward Y. Chen, Francine E. Garrett-Bakelman, Dung-Fang Lee, Benjamin Trinite, Ryan L. Webb, Zichen Wang, Jie Su, Julian Gingold, Ari Melnick, Benjamin A. Garcia, Anthony D. Whetton, Ben D. MacArthur, Avi Ma’ayan, Ihor R. Lemischka

**Affiliations:** ^1^Department of Cell, Developmental and Regenerative Biology, Icahn School of Medicine at Mount Sinai, New York, NY, United States; ^2^Black Family Stem Cell Institute, Icahn School of Medicine at Mount Sinai, New York, NY, United States; ^3^Departament de Biología Cellular, Fisiología i Immunología, Facultat de Biología, Universitat de Barcelona, Barcelona, Spain; ^4^Department of Pharmacology and Systems Therapeutics, Icahn School of Medicine at Mount Sinai, New York, NY, United States; ^5^Stem Cell and Leukaemia Proteomics Laboratory, School of Cancer and Enabling Sciences, Faculty of Medical and Human Sciences, University of Manchester, Manchester, United Kingdom; ^6^Academic Health Science Centre, Wolfson Molecular Imaging Centre, Manchester, United Kingdom; ^7^Centre for Advanced Discovery and Experimental Therapeutics, Central Manchester University Hospitals NHS Foundation Trust, Institute of Human Development, Faculty of Medical and Human Sciences, Manchester Academic Health Science Centre, The University of Manchester, Manchester, United Kingdom; ^8^Department of Molecular Biology, Princeton University, Princeton, NJ, United States; ^9^Department of Medicine, Division of Hematology and Medical Oncology, Weill Cornell Medicine, New York, NY, United States; ^10^Institut de Recerca de La Sida, IrsiCaixa AIDS Research Institute, Germans Trias I Pujol Research Institute, Hospital Universitari Germans Trias I Pujol, Catalonia, Spain; ^11^The Centre for Human Development, Stem Cells and Regeneration, Institute of Developmental Sciences, University of Southampton, Southampton, United Kingdom

**Keywords:** stem cells, epigenetics, multi-omics network, feed forward regulatory loops, proteomics

## Abstract

Cell fate decisions during development are governed by multi-factorial regulatory mechanisms including chromatin remodeling, DNA methylation, binding of transcription factors to specific loci, RNA transcription and protein synthesis. However, the mechanisms by which such regulatory “dimensions” coordinate cell fate decisions are currently poorly understood. Here we quantified the multi-dimensional molecular changes that occur in mouse embryonic stem cells (mESCs) upon depletion of Estrogen related receptor beta (Esrrb), a key pluripotency regulator. Comparative analyses of expression changes subsequent to depletion of Esrrb or Nanog, indicated that a system of interlocked feed-forward loops involving both factors, plays a central part in regulating the timing of mESC fate decisions. Taken together, our meta-analyses support a hierarchical model in which pluripotency is maintained by an Oct4-Sox2 regulatory module, while the timing of differentiation is regulated by a Nanog-Esrrb module.

## Introduction

Understanding how embryonic and induced pluripotent stem cells (ESCs and iPSCs, respectively) regulate cell fate decisions is crucial to realizing their biomedical potential (Liu et al., 2020). The pluripotent state is maintained by intrinsic and extrinsic signals that converge on a network of core transcription factors (TFs) including Oct4, Sox2, and Nanog among others (Boiani and Schöler, 2005; Ivanova et al., 2006; Wang et al., 2006; Chen et al., 2008; Papatsenko et al., 2015; Verneri et al., 2020). These factors are interconnected via transcriptional and protein-protein interactions (Wang et al., 2006; Pardo et al., 2010; van den Berg et al., 2010). However, the exact order of these interactions and the overall molecular mechanisms of the pluripotency network remain unclear.

The diverse phenotypic outputs of the core transcriptional puripotency and self-renewal network are directly or indirectly determined by genetic programs regulated via TF binding to the promoter regions of pluripotency regulators and differentiation inducers and this is mediated through changes in epigenetic states of DNA and chromatin at specific loci. From this point of view, network regulatory functions have clear multidimensionality. While previously many studies were focused on one or two dimensions (e.g., mRNA expression and/or TF binding) at a single time-point, now more studies are starting to address the dynamics of multidimensionality of regulatory networks as they process biological information during cell fate transitions (Lu et al., 2009; Verneri et al., 2020). The goal of the current study is to elucidate global dynamic changes across multiple regulatory dimensions promoted by the depletion of Estrogen related receptor beta (Esrrb), a major core pluripotency TF.

The orphan nuclear receptor Esrrb first emerged as a core pluripotency TF when it was shown that depletion by shRNA results in loss of pluripotency accompanied by differentiation toward epiblast-derived lineages, such as mesoderm and neuroectoderm (Ivanova et al., 2006; Festuccia et al., 2018). Further evidence came from observations that Esrrb can substitute cMyc and Klf4 in iPSC reprogramming (Feng et al., 2009). Interactions of Esrrb with Oct4 and Nanog were demonstrated using genetics and biochemistry. Specifically, Esrrb and Oct4 co-occupy the Nanog proximal promoter and positively regulate its expression (van den Berg et al., 2008). Recent results demonstrating co-binding of Esrrb and Sox2 support this view and suggest that Esrrb is among the key TFs present in ESC while absent in more mature epiblast-derived stem cells (EpiSC) (Hutchins et al., 2013).

Additional studies have shown that the ability of Nanog to confer LIF-independent self-renewal depends on Esrrb and over expression of Esrrb can maintain self-renewal even without Nanog. In addition, Esrrb over expression can recapitulate Nanog activity during iPSC reprogramming (Festuccia et al., 2012). It has also been demonstrated that Esrrb is necessary and sufficient to mediate self-renewal downstream of the Wnt/Gsk3/Tcf3 signaling pathway (Martello et al., 2012). Tcf3 is a transcriptional repressor of pluripotency and is inactivated by Gsk3 in the presence of Wnt signals. Global analyses of Tcf3 target genes pointed to Esrrb as the most likely target TF mediating repression by Tcf3 (Martello et al., 2012). Together, these findings suggest a high position for Esrrb in the hierarchy of pluripotency TFs. Moreover, there appears to be at least partial functional redundancy between Esrrb and Nanog mediated by a highly interconnected regulatory module in which Esrrb and Nanog are linked by protein–protein as well as transcriptional interactions (Loh et al., 2006; Wang et al., 2006; van den Berg et al., 2008, 2010; Zhang et al., 2008).

Global molecular changes following shRNA-mediated depletion of Nanog were previously described by our group (Lu et al., 2009). Here, we expand these analyses for Esrrb depletion by considering five regulatory dimensions: (i) dynamics of global mRNA, (ii) dynamics of promoter methylation, (iii) dynamics of the nuclear proteome, and dynamics of (iv) activating (H3K4me3) as well as (v) repressive (H3K27me3) histone marks and provide a comprehensive view of evolving cell fate changes during the exit of the pluripotent state.

Meta analyses of the five regulatory dimensions and superposition of the current Esrrb and previous Nanog datasets (Ivanova et al., 2006; Loh et al., 2006; Lu et al., 2009; Macarthur et al., 2012), reveal several topological elements including network motifs linking Esrrb and Nanog with other core pluripotency TFs and their targets. Commonly identified network motifs such as feed-forward (FFL) or bi-parallel loops (BPLs) involve both TF/target interactions. In general, FFL are known to be widely present in biological networks (Milo et al., 2002). Along with the structural analysis of the pluripotency gene regulatory network architecture (PGRN), we have found a hierarchical model in which pluripotency is maintained by an Oct4-Sox2 regulatory module, while the timing of differentiation is regulated by a Nanog-Esrrb.

## Materials and Methods

### ES Cell Culture

The murine ESC lines with controllable Esrrb expression (Esrrb_R) or controllable Nanog expression (Nanog_R) were constructed and characterized previously (Ivanova et al., 2006), and were maintained as described on irradiated primary mouse embryonic fibroblasts (MEFs). For all experiments, ESCs were cultured on 0.1% gelatin-coated tissue culture plates without feeder cells. To induce differentiation, we withdrew Doxycycline (Dox) (1 μg ml-1, Sigma) from the media while maintaining all other routine ESC nutrients: D-MEM–High Glucose (Dulbecco’s modified Eagle’s medium-1X-High Glucose) (Gibco, Invitrogen), 15% FBS (fetal bovine serum) (Hyclone, Thermo Fisher Scientific), 100 mM MEM non-essential amino acids, 0.1 mM 2-mercaptoethanol, 1 mM L-glutamine, Penicillin/Streptomycin (Gibco, Invitrogen) and 10^3^ U ml^–1^ LIF (Chemicon, Millipore). All cell cultures were maintained at 37°C with 5% CO_2_ and cells were plated at a density of 3 × 10^5^ cells per 10 cm dish.

### Microarray Gene Expression Profiling

RNA probes from each time point were hybridized to Affymetrix Gene Chip Mouse Gene 1.0 ST microarrays (three biological replicates: 12 arrays in total) according to the manufacturer’s protocols by the Genomics Core facility at the Icahn School of Medicine at Mount Sinai, New York. Data were normalized using the Robust Multichip Average (RMA) method in the Affymetrix Expression Console software. Expression measurements were obtained by taking the mean readings for gene-specific probe sets and the data were log2 normalized. Microarray data analysis is described at the Supplemental Experimental Procedures file.

### Array-Based Methylation Analysis Using HELP

The HELP assay was performed as previously described (Khulan et al., 2006). We used the HELP mouse promoter array MM9_HX3 on a 720K platform which is designed to cover 117,000 *Hpa*II amplifiable fragments (genomic sequences between two flanking *Hpa*II sites 200–2000 bp apart) within CpG islands and promoters of the well characterized RefSeq genes derived from the UCSC RefFlat files. Hybridization was performed at the Weill Cornell Medical College Epigenomics Core Facility. Scanning was performed using a NimbleGen MS 200 scanner. For array-based methylation data analyses see Supplementary Table 4, which contains the genes that significantly changed their methylation state in the Esrrb and Nanog time series.

### Chromatin Immunoprecipitation

Chromatin immunoprecipitations (ChIPs) were performed as described (Boyer et al., 2005). Images acquired from the Solexa sequencer were processed through the bundled Solexa image extraction pipeline and aligned to the Mouse July 2007 assembly (NCBI37/mm9) using ELAND software. Supplementary Figure 1E shows the specificity of the anti-Esrrb antibody used for ChIP and Supplementary Figure 1F show the enrichment of the Esrrb tags relative to the TSS.

### ITRAQ

Nuclear protein samples were prepared using a previously described method (Hsu and Hung, 2007; Supplementary Figure 1G). Proteomic measurements were performed as follows. Samples from the four time points (day 0, and days 1, 3, and 5 after Dox removal) were labeled using eight channel isobaric tagging reagents [isobaric tag for relative and absolute quantification (iTRAQ), Applied Biosystems] according to the published protocol (Unwin et al., 2010). Data analyses were performed using ProteinPilot v3 software (AB Sciex). Only proteins detected with a minimum of two or more high-scoring spectra (likelihood *p*-value < 0.005) were accepted. The combined search results led to a set of 1790 high-confidence protein identifications (Supplementary Figures 1H,I).

## Results

### Meta-Analysis of Global Molecular Changes Resulting From Esrrb Removal

**FIGURE 1:**
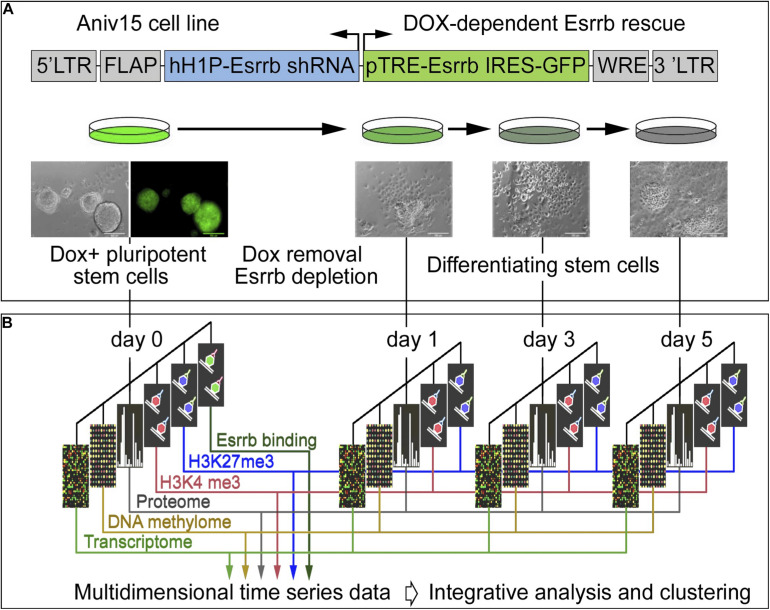
Experimental strategy and data integration. **(A)** Schematic representation of transgenic construct used to deplete Esrrb in order to promote differentiation of mESCs. Lentiviral vector for conditional expression of Esrrb is shown on the top (Ivanova et al., 2006). Endogenous Esrrb is depleted with short hairpin (sh) RNA and complemented by shRNA “immune” version of Esrrb expressed in a doxycycline (Dox)-dependent manner. Removal of Dox results in downregulation of the exogenous Esrrb leading to differentiation. FLAP is a nucleotide segment that improves transduction efficiency; hH1P-Esrrb is the endogenous Esrrb specific shRNA cassette (in blue); pTRE-Esrrb is GFP-tagged exogenous Esrrb cassette (in green); WRE is the woodchuck hepatitis virus post-transcriptional regulatory element. **(B)** Experimental time course, analyzed data types and data integration strategy. At the initial day 0 time point, Esrrb is expressed in the presence of Dox; at day 1, 3, and 5 time points Esrrb is downregulated following removal of Dox. Each data type (epigenetic, transcriptional, mRNA, and proteomic) is collected at each time point and integrated into a single multi-dimensional time-series data set (See also Supplementary Figure 1 for time series controls).

**FIGURE 2:**
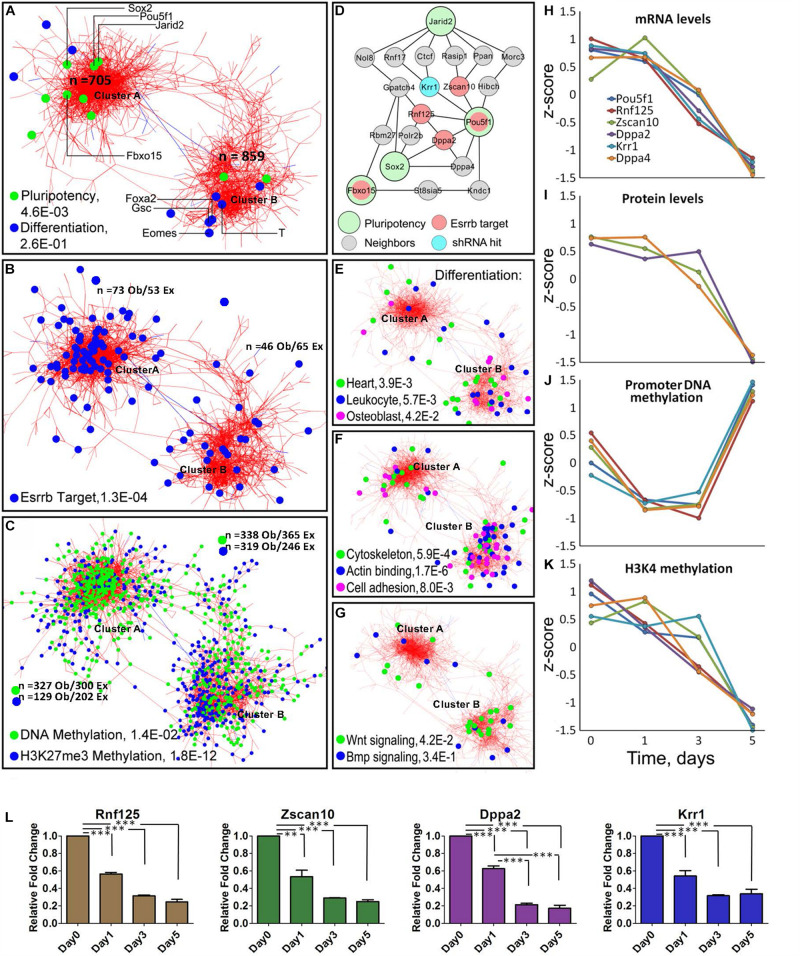
Meta-analysis of differentiation reveals Oct4-Sox2 activation domain. **(A)** Co-expression network constructed from time-series data encompassing 1563 genes/gene-products and five types of dynamic measurements (levels of mRNA, protein, promoter DNA methylation, H3K4me3, and H3K27me3). Each node represents a gene/gene product, and the edges connect those gene/gene products whose expressions are similar across the five regulatory layers analyzed. The network shows the presence of two global clusters, cluster A represented by pluripotency genes (*P* = 4.6E-0.3) and cluster B represented by differentiating genes (*P* = 2.6E-0.1) according to the ESCAPE database (Xu et al., 2013). Green dots represent well-known pluripotency gene/gene products and blue dots represent differentiated gene/gene products, respectively as examples. **(B)** Distribution of Esrrb target genes in the network shows that the majority are localized in cluster A. **(C)** Genes with changing promoter DNA methylation levels (green nodes) are highly represented in cluster A (Supplementary Figure 2 shows their change toward a hypermethylated state by day 5). In contrast, genes with changing H3K27me3 levels (blue nodes) are preferentially localized cluster B (Supplementary Figure 2 shows the erase of this mark by day 5). **(D)** The largest interconnected subnetwork, containing pluripotency genes and their direct neighbors (see location of Pou5f1 cluster A in panel **A**). Pluripotency “seed” genes are represented by large circles, direct Esrrb targets are in red and a hit from a high-content shRNA screen is in blue. Oct4 occupies the central position in the network with the majority of Esrrb target genes as close neighbors. **(E–G)** Distribution of selected GO terms in cluster A and cluster B. Only GO terms significantly enriched in each of the two clusters were considered using the gene set enrichment web server Enrichr (Kuleshov et al., 2016). Genes present in cluster B show enrichment in cytoskeleton, actin binding, cell adhesion, and Wnt signaling categories. **(H–K)** Dynamic expression profiles for gene/gene-products included in the network **(D)**. Four types of data (mRNA, protein, promoter DNA methylation, and H3K4me3) are shown. Dynamic changes within each regulatory layer are in good agreement among all genes. **(L)** Validation of predicted expression levels for Rnf125, Zscan10, Dppa2, Krr1; gene expression changes were measured by qRT-PCR. All data are represented as mean ± SD; *n* = 3 and *p* < 0.05 (one-way ANOVA and Bonferroni’s post-test). ^∗∗^*P* < 0.01, ^∗∗∗^*P* < 0.001.

In order to explore the function of Esrrb and the Nanog/Esrrb module we analyzed genome wide epigenetic, transcriptional and post-transcriptional processes regulated by Esrrb. For this study, we utilized a lentiviral/shRNA-based genetic complementation system to deplete Esrrb under serum/Lif conditions (Ivanova et al., 2006; Lee et al., 2012; Figure 1A and see Supplementary Figures 1A–D for controls of the differentiation process). We measured temporal changes for five regulatory layers (Figure 1B) including: (1) mRNA levels (microarrays), (2) promoter DNA methylation patterns (*Hpa*II tiny fragment Enrichment by Ligation-mediated PCR, HELP-assay) (Khulan et al., 2006), (3) nuclear proteins (isobaric tag mass spectrometry, iTRAQ) (Unwin et al., 2010), (4) genome-wide H3K4me3 (chromatin immunoprecipitation coupled with DNA sequencing, ChIP-seq), and (5) genome wide H3K27me3 ChIP-seq modification levels. We also identified (6) direct Esrrb target genes (ChIP-seq) (Supplementary Figures 1E,F). Analyses 1–5 were performed at days 0, 1, 3, and 5 after depletion of Esrrb while analysis 6 was only performed at day 0. To obtain an integrated, multi-dimensional view of global regulatory changes triggered by the loss of Esrrb we have constructed a co-expression multi-omics network based on changes observed relative to day 0 for each regulatory layer: transcriptome, proteome, methylome, H3K4me3, and H3K27me3 (details in Supplemental Experimental Procedures and Supplementary Table 1). Results of the integrative analysis through a co-expression distance matrix across all five regulatory layers during the 5 day time course (Figure 1B) are shown in the Figure 2 using Cytoscape tools (Otasek et al., 2019), whereas visualization of the dynamic changes in the network for each regulatory layer are shown in Supplementary Movie 1 and Supplementary Figure 2 using the GATE software (Macarthur et al., 2009).

Interestingly, using Cytoscape (Otasek et al., 2019) we obtained a co-expression multi-omics network across the different regulatory layers with a cut-off of *p* < 10^–6^, which contained two major clusters; one where there is high representation of pluripotency genes (cluster A) and another highly represented by differentiated genes (cluster B) according to the Embryonic Stem Cell Atlas from Pluripotency Evidence (ESCAPE) database (Xu et al., 2013). Analyses of cluster A (Figure 2A) and its highly interconnected core (Figure 2D) revealed significant downregulation of the essential components of the core pluripotency network (Figure 2L). The core network contains the major pluripotency factors Oct4 and Sox2 closely linked to the other established pluripotency factors such as Krr1 (You et al., 2015), Dppa2 and Dppa4 (Hernandez et al., 2018), and Zscan10 (Yu et al., 2009), a known transcriptional regulator of Oct4.

Most genes within the identified Oct4-Sox2 network module, remarkably displayed similar dynamics across all molecular layers (Figures 2H–K) showing tight coordination of pluripotency genes across different epigenetic dimensions (or regulatory layers) in the Oct4-Sox2 network. This strong correlation suggests a possible mechanism of how cells maintain the pluripotency state via direct positive feedback loops.

Analysis of the methylation patterns across the clusters notably showed a greater number of genes associated with dynamic changes toward a hypermethylated state in cluster A represented by pluripotency genes than in cluster B are represented by differentiation genes (Supplementary Figure 2 global promoter methylation layer over time and Supplementary Movie 1). In contrast, higher numbers of genes in cluster B were associated with the downregulation in H3K27me3 levels (Figure 2C and Supplementary Figure 2 H3K27me3 layer and Supplementary Movie 1). A clear example of this pattern was found within the core network, extracted from the pluripotency cluster based on the known pluripotency markers (Figure 2D). In this network, genes linked to the Oct4-Sox2 domain (Pou5f1, Dppa4, Rnf125, Zscan10, and Krr1) were also associated with changes in promoter methylation, but not with changes in H3K27me3 levels (Figure 2J and Supplementary Figure 3A). This suggests that promoter methylation could be the immediate response for shutting-down pluripotency genes as the majority of the gene promoter’s, transit from a hypomethylated state toward a hypermethylated state by day 5, especially in the cluster A where we observed more genes related to pluripotency (Supplementary Figure 2). A separate analysis of the global methylome also corroborate that the majority of the gene’s promoters (even higher fraction than in the case of Nanog depletion) undergo transition towards a hypermethylated state by day 5 (Supplementary Figure 3B). Pairwise analysis of mRNA and protein level dynamics has shown high coherence of mRNA and protein expression levels for most pluripotency genes (Supplementary Figure 4A and Supplementary Movie 2). A majority of Esrrb target genes were found to be associated with cluster A (Figures 2B,D and Supplementary Table 2). Gene Ontology (GO) analyses of the two clusters using Enrichr (Kuleshov et al., 2016) showed enrichment for cytoskeleton, actin binding and cell adhesion categories in cluster B. Enrichment of Wnt signaling components was also evident in cluster B, suggesting that depletion of Esrrb promotes the activation of this pathway (Figures 2E–G).

### Synergistic and Unique Functions of Esrrb and Nanog in Pluripotency and Differentiation

**FIGURE 3:**
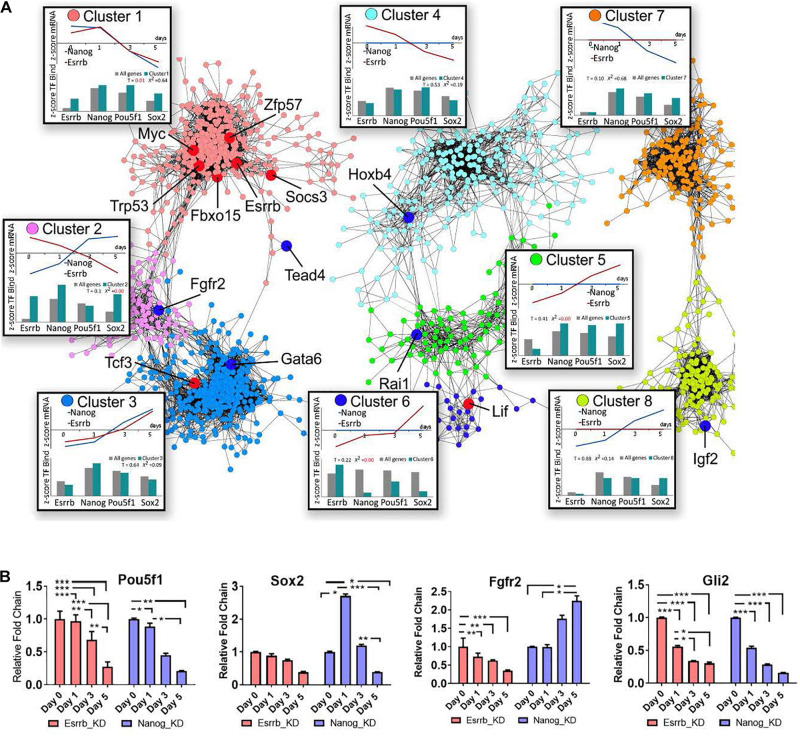
Co-expression map for gene responses to depletion of Nanog or Esrrb. **(A)** Co-expression network based on time-series data collected after Nanog or Esrrb downregulation. Nodes in the network are color-coded according to the identified co-expression clusters; large nodes in red mark pluripotency genes, while large nodes in dark blue mark differentiation genes according to the ESCAPE database (Xu et al., 2013) (gene names from each specific cluster are shown in Supplementary Table 3). Boxes next to each co-expression cluster show as line plots, the normalized (*z*-score) average expression profiles across all genes within the cluster over the time series. Histograms in the bottom of the boxes, show the *z*-score average TF binding profiles for the clustered genes. The histogram compares the fraction of known ChIP targets for a given TF in a cluster versus that expected based on entire genome. (*x*^2^ = 0 when the observed and expected values are equal) (*T*-value higher than 1 means the difference is significative.) Three disconnected network domains correspond to genes with changing expression in both Nanog and Esrrb datasets (clusters 1–3), genes with changing expression only in the Esrrb dataset (clusters 4–6) and genes with changing expression only in the Nanog dataset (clusters 7 and 8). **(B)** Experimental validation of gene responses predicted from panel **(A)**. Expression levels of Oct4, Sox2, Fgfr2, and Gli2 measured by qRT-PCR. All data are represented as mean ± SD; *n* = 3 and *p* < 0.05 (one-way ANOVA and Bonferroni’s post-test). ^∗^*P* < 0.05, ^∗∗^*P* < 0.01 and ^∗∗∗^*P* < 0.001.

To characterize the specific roles of Esrrb and Nanog in the pluripotency network, we constructed a co-expression gene network (Otasek et al., 2019) based on our Esrrb and previously published Nanog time-series depletion data for transcriptome (Lu et al., 2009) and identified clusters with characteristic expression and TF binding properties (Figure 3A, Supplementary Movie 3, and Supplementary Table 3). Given that many genes in the integrated Esrrb-Nanog data set (1615 significantly changing genes) were targets of more than one TF, we included in the analyses the *in vivo* binding patterns of the four master pluripotency regulators (Oct4, Sox2, Nanog, and Esrrb) (Chen et al., 2008; Kim et al., 2008; Marson et al., 2008).

The co-expression network based on the Nanog and Esrrb gene expression changes revealed eight major clusters. Based on the distribution of pluripotency markers (Figure 3A, genes marked in red across the different clusters), cluster 1 contained mostly pluripotency genes based on the ESCAPE database (Xu et al., 2013). Genes in this cluster were strongly downregulated in response to depletion of either Esrrb or Nanog. Analysis of TF binding has shown that most genes in cluster 1 are targets of all four major pluripotency TFs (Esrrb, Nanog, Oct4, and Sox2, see the box “cluster 1” in the Figure 3A). In contrast, cluster 3 represents differentiation-specific genes upregulated by day 5; genes in clusters 1 and 3 respond to both Esrrb and Nanog depletion in a coherent manner. However, it is interesting to note that the average TF binding pattern in cluster 3 is largely opposite to that in the pluripotency cluster 1. Targets of Esrrb, Oct4 and Sox2 are underrepresented in cluster 3 (see box labeled cluster 3), while targets of Nanog are slightly overrepresented. This suggests that Nanog has a major repressive function when bound to many differentiation genes. This fact was also revealed when we performed the analysis of promoter methylation for both Nanog an Esrrb knockdown time-series. In the case of Nanog removal, we observed more genes losing promoter methylation marks (hypomethylation) than in the case of Esrrb removal (Supplementary Figure 3B and Supplementary Table 4). Cluster 2 is the most interesting because it contains genes that are conversely expressed after depletion of Nanog or Esrrb; thereby illustrating specific functions for the two pluripotency regulators. According to the averaged expression profiles, many genes in this group are activated by Esrrb and repressed by Nanog (but not vice versa). The TF binding profiles in cluster 2 show moderate over representation of all pluripotency TFs, with the exception of Oct4.

Interestingly, the Fgf signaling pathway receptor Fgfr2, was identified among the genes that are conversely expressed after depletion of Esrrb or Nanog (cluster 2). Genomic regions at Fgfr2 promoter contain binding sites for all four TFs considered in this study (Esrrb, Nanog, Oct4, and Sox2).

Further inspection of the co-expression map revealed that genes in cluster 4 are downregulated only in the absence of Esrrb but are mainly regulated by Oct4 strengthening a close interaction between Esrrb and Oct4 in the regulation of this subset of genes. Cluster 5 contains genes upregulated in the absence of Esrrb, which are mostly regulated by Oct4, Sox2, and Nanog but not by Esrrb. In contrast, cluster 6 represents genes upregulated upon removal of Esrrb that are targeted largely by Esrrb itself. This shows that Esrrb not only plays a role as a major pluripotency factor but also has important functions during late differentiation. Finally, clusters 7 and 8 contain genes selectively responding to Nanog, but not Esrrb depletion; genes outside the eight co-expression clusters show no responses to depletion of Esrrb or Nanog and no significant binding by the pluripotency TFs (data not shown). Figure 3B shows experimental RT-qPCR validation of some pluripotency genes as well as some genes that respond to depletion of Esrrb or Nanog.

### Predicted Properties of Network Motifs Linking Esrrb and Nanog With Their Targets

**FIGURE 4:**
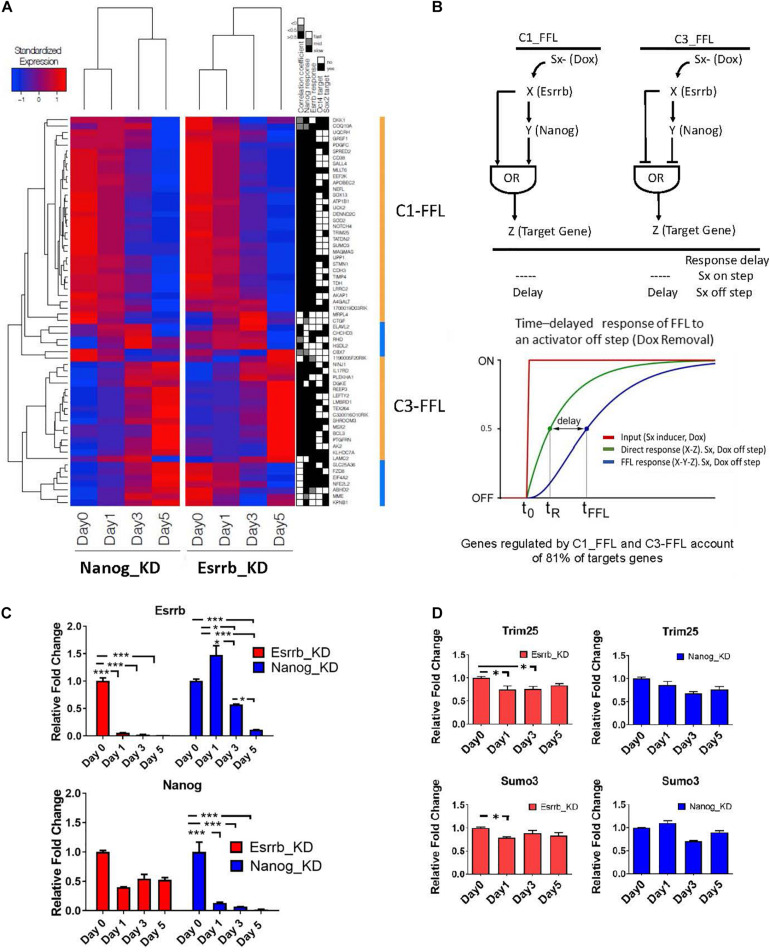
Predicted time delay responses in the Esrrb-Nanog domain. **(A)** Hierarchical clustering of expression changes of Esrrb and Nanog targets during both downregulation time courses. The black, gray and white map on the right show’s classification of the target gene responses as fast, medium or slow, respectively; the fourth and fifth columns mark direct targets of Oct4 and/or Sox2, respectively. **(B)** Structure of the coherent type 1 (C1-FFL) and type 3 (C3-FFL) feed-forward loops with an OR logic. Transcription factor X regulates transcription factor Y, and both regulate a target gene, Z. Sx is the inducer signal that can be ON or OFF. Graph of the time delay response of FFL to the OFF inducer signal step **(C)** Gene expression levels measured by qRT-PCR of Esrrb (upper graph) and Nanog (lower graph) following Esrrb (red graph) or Nanog (blue graph) depletion. All data are represented as mean ± SD; *n* = 3 and *p* < 0.05 (one-way ANOVA and Bonferroni’s post-test). ^∗^*P* < 0.05, ^∗∗^*P* < 0.01, ^∗∗∗^*P* < 0.001. **(D)** Experimental validation of the delayed or “slow” predicted responses for the C1-FFL. Expression levels of, *Trim25* and *Sumo3* measured by qRT-PCR following Esrrb depletion (red graph) or Nanog depletion (blue graph). All data are represented as mean ± SD; *n* = 3 and *p* < 0.05 (one-way ANOVA and Bonferroni’s post-test). ^∗^*P* < 0.05.

Since we observed that, many genes coherently respond to both Esrrb and Nanog upon an external stimulus (Doxycycline removal), we focused our analysis on exploring potential FFL motif types linking these two regulators, as these kind of motifs need a respond to external stimuli. FFL motifs commonly occur in biological networks and play important functions in many regulatory pathways (Shen-Orr et al., 2002; Mangan and Alon, 2003; Goentoro et al., 2009; Papatsenko and Levine, 2011). Each FFL has a unidirectional structure consisting of three nodes: an upstream regulator X that regulates a downstream regulator Y, which in turn regulates a downstream target Z. An additional edge is directed from X to Z, thus closing a unidirectional “loop” (Figure 4B). Each interaction can be suppressing or activating, resulting in eight distinct FFL structures (Mangan and Alon, 2003). Here we have considered FFLs incorporating Esrrb (X), Nanog (Y), and their potential target genes (Z). Analysis of transcriptional changes classified in fast, middle, and slow responses upon depletion of Esrrb or Nanog showed 2 different structural types of FFL motifs, the coherent type I FFL (C1-FFL) and the coherent type 3 FFL(C3-FFL) from the possible eight types when either Esrrb or Nanog is knockdown (Mangan and Alon, 2003; Figure 4A). Interestingly, coherent FFLs of types 1 (C1-FFL) and 3 (C3-FFL) account for regulation of the majority (81%) of the Esrrb-Nanog target genes (Figures 4A,B) and all genes presented a decay or growth in expression in a slow time scale.

One interesting property of the coherent FFLs is delay to upstream stimuli. If expression of a downstream target gene (Z) requires expression of both upstream regulators (X, Y) but only one of the two responds to an input signal (Doxycycline), the expression of the second regulator causes an expression delay or a slow response (Mangan and Alon, 2003). This effect is shown schematically in Figure 4B. In this context, the “signal” (removal of Doxycycline) causes the downregulation of Esrrb or Nanog expression respectively in each time course where either the Esrrb-R or the Nanog-R rescue clone were used, and dynamics of genes downstream of Esrrb and Nanog provided a measurable “response” from which putative regulatory logic can be inferred. Notably, as Nanog levels are not completely abolish upon Esrrb depletion (Figure 4C), this implies a certain delay in the downregulation of Esrrb-Nanog target genes (Figure 4A). This delayed response, for a certain group of genes, is what we observed in each time series experiments with either (Esrrb-R) or (Nanog-R) cell lines, which is characteristic of coherent FFL regulation with an “OR” logic when the input signal is off (Mangan and Alon, 2003).

Experimental validation of the delayed or slow predicted responses from genes of the C1-FFL1 type was carried out using RT-PCR (Figure 4D). The delayed or slow response effect can be observed in the downregulation of the gene Sumo3 and Trim25 (Figure 4D) in comparison with the dynamics observed in other genes like Gli2 (Figure 3B), not participating in this type of motif as only Esrrb binds its promoter but not Nanog (Supplementary Table 2).

From this analysis we conclude that time delay responses in the Esrrb-Nanog domain may provide a temporal window necessary for network information processing and proper response to signals incoming via Esrrb or Nanog.

### Position of the Esrrb-Nanog Module in the Transcription Regulatory Hierarchy of mESC

**FIGURE 5:**
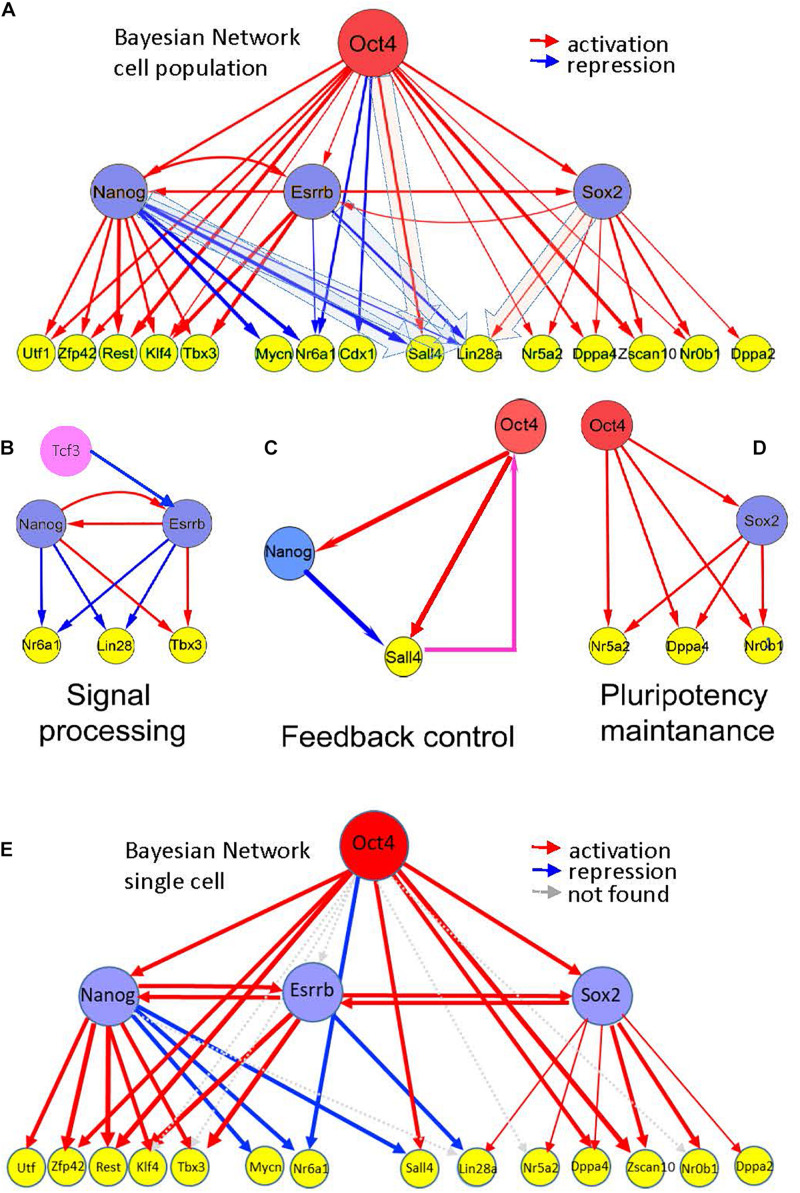
Hierarchy of pluripotency gene networks. **(A)** A Bayesian pluripotency network constructed based on knockdown (and/or knockout) differentiation studies in combination with *in vivo* TF binding analyses (Supplementary Table 5, fraction of predicted targets is shown). The red arrows show activating and the blue arrows show repressive links between the genes. Edge thickness represents the level of experimental support based on all analyzed independent data sources (Supplementary Table 5). Esrrb is tightly linked with other core pluripotency factors, including Nanog and Sox2. **(B–D)** Emerging information-processing circuits in the context of the gene network shown in **(A)**. **(B)** Nanog-Esrrb module, responsible for interpretation and processing external signals, such as Gsk3/Tcf signaling, targeting Esrrb and Nanog, or Lif signaling targeting Nanog and Klfs (Lif is not shown), Klf4 is shown on the panel **(A)**. The Nanog-Esrrb module largely contains multiple coherent feed-forward loops. **(C)** Feedback control circuits, such as Oct4-Nanog-Sall4 (see the panel **A**) are responsible for controlling Oct4 concentration by the downstream pluripotency genes (shown in a box); largely represented by incoherent feed-forward loops with a feedback. Oct4 regulation by Sall4 is suggested by several recent studies (Buganim et al., 2012). **(D)** Pluripotency maintenance unit, Oct4 and Sox2 synergistically activate most of pluripotency genes, this unit also consists of coherent feed-forward loops. **(E)** Bayesian pluripotency network based on single cell data in combination with *in vivo* TF binding analyses. The red arrows show activating and the blue arrows show repressive links between the genes. Gray arrows depict absent connections with respect to the whole cell population network from Figure 5A. Cdx1 was not analyzed in the single cell data.

Based on the Esrrb time course data from this study, we explored the position of the Esrrb/Nanog module within the mESC PGRN. To account for a hierarchical model that governs the pluripotent state we integrated data from the present study with retrieved genomic binding data (Chen et al., 2008; Kim et al., 2008; Marson et al., 2008; Martello et al., 2012) and expression data after depletion of the main pluripotency TFs Oct4 (Ivanova et al., 2006; Loh et al., 2006; Matoba et al., 2006), Sox2 (Ivanova et al., 2006), Nanog (Ivanova et al., 2006; Loh et al., 2006; Lu et al., 2009; Macarthur et al., 2012), and Esrrb (Ivanova et al., 2006; Feng et al., 2009; and the present study). A Bayesian transcriptional network has been reconstructed based on immediate responses (1 or 2 days) following depletion of a given TF. Such analyses identified genes that respond quickly to concentration changes of a potential upstream regulator and thus, are more likely to represent direct transcriptional targets. Supplementary Table 5 shows the most probable TF targets, ranked according to the level of significance. From these data, high confidence targets of Esrrb are Sox2 and Nanog occupying ranks 12 and 23 among all TFs (1578 murine TFs were taken into account). This represents the 0.8 and 1.5% top percentiles, respectively. Even more strikingly, Esrrb occupies rank 2 among Nanog targets as well as among targets of Sox2. Among Oct4 targets Esrrb occupies rank 25, higher than Sox2 (rank 64) and Nanog (rank 108). These data strongly supported the existence of mutual interactions between Esrrb/Nanog and Esrrb/Sox2 (Figure 5A). The overall architecture of the reconstructed network suggests that Esrrb is tightly linked to the other core pluripotency TFs and occupies the central position in the transcriptional hierarchy. Esrrb is also close to Nanog and Klf4, which appears to comprise the Nanog-Esrrb module, responsible for the processing of incoming external signals (Figure 5B). Interestingly, properties of this structural domain are the time-delayed responses as we have shown in Figure 4.

Several other key pluripotency factors were not found among the immediate targets of the major core factors (Oct4/Sox2/Nanog/Esrrb). For example, the Tcf3 repressor, acting upstream of Nanog and Esrrb and mediating external signals, such as Wnt was not found, thus supporting its possible regulation by and alternative or independent network from the one regulated by Oct4, Nanog, Esrrb, and Sox2 (not shown in Figure 5). A second example is cMyc a TF expressed in ESCs (Supplementary Table 5). This oncogenic TF is a point of intersection between the pluripotency and the cancer transcriptional networks (Kim et al., 2010).

Inspection of the reconstructed transcriptional mESC hierarchy revealed downstream genes Sall4 and Lin28a, which were alternatively regulated by the core TFs. These genes are positively regulated by Oct4 (Sall4) and Sox2 (Lin28a) but are negatively regulated by Nanog (Sall4 and Lin28a) and Esrrb (Lin28a). This interesting mode of regulation, along with the previously reported feedback link between Sall4 and Oct4 (Buganim et al., 2012; Figure 5C) suggest these TFs (Esrrb and Nanog) as candidates that trigger state switching of the entire PGRN through a feedback regulatory mechanism. Interestingly, the Oct4-Sox2 domain of the reconstructed hierarchy matched the core network reconstructed from our multi-omics analysis (compare Figures 2D, 5D); thus, suggesting the existence of yet another structural network domain in the pluripotency network.

Finally, we integrated mESC single cell expression profiles into our hierarchical model (Figure 5E) to compare with the whole population previously analyzed (Figure 5A) and the results showed a high degree of conservation. 78% of the activating or repressing interactions were preserved.

## Discussion

The key events that yield cell fate decisions occur over time as regulatory networks process biological information. In fact, it is likely that actual changes in cell phenotype may be emergent properties of collective network dynamics (Lu et al., 2009; Kim et al., 2020). Network dynamics and network-mediated information processing remain largely unexplored in most biological systems. Here, we apply an integrated multi-level and temporal experimental/computational approach for dynamic network analyses. We acquire and integrate epigenetic, mRNA and protein datasets from mESCs undergoing cell fate changes in response to a single, well-defined perturbation, the shRNA-mediated depletion of Esrrb, a key pluripotency TF. Our studies provide a comprehensive view of biological information processing mechanisms as pluripotent stem cells exit the pluripotency state.

Analysis of the dynamic changes in gene product levels and epigenetic modifications revealed two major clusters suggesting two major states of the regulatory mESC gene network in the presence of serum plus Lif conditions (Figure 2A). Genes in the first cluster are shut down upon the exit from pluripotency, while genes in the second cluster are activated upon differentiation. More complex patterns of behavior (e.g., intermediate transition states) were not detected; however, the differentiation cluster contains certain sub clusters, potentially reflecting the presence of alternatively differentiating cell lineages (Figures 2E–G).

The integrated multi-omic analyses based on co-expression among the regulatory layers suggest that different epigenetic levels contribute unequally to the exit from pluripotency and initiation of differentiation. Specifically, dynamic changes in the promoter methylation were tightly associated with the core of the pluripotency cluster, suggesting this is the major mechanism for shutting-down pluripotency genes (Figure 2C). In contrast, dynamic changes associated with the repressive H3K27me3 histone methylation mark were largely observed in the differentiation cluster but not the pluripotency cluster. Although this observation might reflect the abundance of CpG rich domains at promoters, it is also possible that the alternative levels of regulation are respectively more effective in erasing epigenetic memory and facilitating alternative cell fate commitment directions upon exiting pluripotency.

Overall, the data suggests tight functional linkage between Esrrb and Nanog TFs (Figure 4A), which might be essential for processing external and internal signals. Network reconstruction studies also support the existence of the Esrrb-Nanog domain in the context of the pluripotency network (Figure 5B).

Based on integrative analysis of multiple knockdown studies and *in vivo* binding assays, we constructed a hierarchical model describing interactions between the major TFs upon differentiation (Figure 5). Major features of the established hierarchy included close linkage between the Esrrb and Nanog and the presence of incoherent feed forward loops (iFFL), incorporating the major core pluripotency factors and some of their target genes. Both Nanog and Esrrb were found to be the parts of a BPL targeting *Lin28a*, Nanog was a part of FFL targeting *Sall4* (Figure 5C).

Such incoherent FFLs and BPLs are known to be broadly involved in developmental pattern formation, cell fate specification and many other biological processes, which require switching of gene activity states and establishing threshold concentrations.

Another interesting feature found in our reconstructed hierarchy network is a noticeable functional difference between the Esrrb-Nanog and Oct4-Sox2 network domains. The former pair is involved in both activation and repression (Figure 5B, blue and red arrows), while the latter serves mainly as a pair of activators (Figure 5D, red arrows). The “general” activation functions of the Oct4-Sox2 pair seem to maintain the expression of pluripotency genes, while the functions of Esrrb-Nanog pair more likely contribute to information processing and decision-making.

This presence of the Oct4-Sox2 and Esrrb-Nanog modules is also supported by systematic knockdown studies of 100 TFs (KD-100) in mESC where the Oct4-Sox2 module maintains pluripotency and prevents extraembryonic fates while Nanog-Esrrb and SallL4 coordinate different signal processes preventing cell fate differentiation (Nishiyama et al., 2013). In particular, Sall4 plays a clear feedback control in the pluripotency maintenance as there is evidence that *Sall4* is not only regulated by the core factors it also provides feedback control to the system by regulating *Oct4* (Figure 5C; Yang et al., 2010).

Finally, to validate our hierarchical model at the single cell level, we analyzed single cell gene expression profiles of mESCs (Papatsenko et al., 2015). To our surprise, 78% of the interactions observed in the cell population were maintained at the single cell level (see Figure 5E).

It has been shown that iPSC reprogramming is a stochastic process where early expression of Esrrb, Utf1, Lin28, and Dppa2 are good predictors for cells that will eventually yield fully reprogrammed iPSCs, expressing high levels of Oct4 and Sox2 (Buganim et al., 2012, 2014). To a certain degree, the reprogramming process is opposite (reversed) to differentiation; therefore, one may expect that the reprogramming hierarchy would be opposite to that in differentiation. Indeed, factors predicting successful iPSC reprogramming occupy lower positions in our hierarchical network, while the core factor Oct4 occupies the highest position. Such comparison of the differentiation and the reprogramming networks suggests a high degree of flexibility of the pluripotency network and its potential for dynamic rewiring.

## Conclusion

In summary, our results suggest flexibility in the architecture of the pluripotency network incorporating at least two large network domains (Esrrb-Nanog and Oct4-Sox2) and numerous feed-forward as well as feedback regulatory interconnections that collectively control cell fate transitions in pluripotent stem cells.

## Data Availability Statement

The datasets presented in this study can be found in online repositories. The names of the repository/repositories and accession number(s) can be found in the article/Supplementary Material.

## Author Contributions

AS designed the project, performed the experiments, and prepared the manuscript. AV, RU, GL, FG-B, D-FL, JS, and AW performed the experiments. DP, AvM, HX, EC, BT, RW, ZW, and RU performed the bioinformatics analyses. IL prepared the manuscript. All authors reviewed and approved the manuscript.

## Conflict of Interest

The authors declare that the research was conducted in the absence of any commercial or financial relationships that could be construed as a potential conflict of interest.

## References

[B1] Boiani M. Schöler H. R. (2005). Regulatory networks in embryo-derived pluripotent stem cells. *Nat. Rev. Mol. Cell Biol.* 6 872–884. 10.1038/nrm1744 16227977

[B2] Boyer L. A. Tong I. L. Cole M. F. Johnstone S. E. Levine S. S. Zucker J. P. (2005). Core transcriptional regulatory circuitry in human embryonic stem cells. *Cell* 122 947–956. 10.1016/j.cell.2005.08.020 16153702 PMC3006442

[B3] Buganim Y. Faddah D. A. Cheng A. W. Itskovich E. Markoulaki S. Ganz K. (2012). Single-cell expression analyses during cellular reprogramming reveal an early stochastic and a late hierarchic phase. *Cell* 150 1209–1222. 10.1016/j.cell.2012.08.023 22980981 PMC3457656

[B4] Buganim Y. Markoulaki S. Van Wietmarschen N. Hoke H. Wu T. Ganz K. (2014). The developmental potential of iPSCs is greatly influenced by reprogramming factor selection. *Cell Stem Cell* 15 295–309. 10.1016/j.stem.2014.07.003 25192464 PMC4170792

[B5] Chen X. Xu H. Yuan P. Fang F. Huss M. Vega V. B. (2008). Integration of external signaling pathways with the core transcriptional network in embryonic stem cells. *Cell* 133 1106–1117. 10.1016/j.cell.2008.04.043 18555785

[B6] Feng B. Jiang J. Kraus P. Ng J. H. Heng J. C. D. Chan Y. S. (2009). Reprogramming of fibroblasts into induced pluripotent stem cells with orphan nuclear receptor Esrrb. *Nat. Cell Biol.* 11 197–203. 10.1038/ncb1827 19136965

[B7] Festuccia N. Halbritter F. Corsinotti A. Gagliardi A. Colby D. Tomlinson S. R. (2018). Esrrb extinction triggers dismantling of naïve pluripotency and marks commitment to differentiation. *EMBO J.* 37:e95476. 10.15252/embj.201695476 30275266 PMC6213284

[B8] Festuccia N. Osorno R. Halbritter F. Karwacki-Neisius V. Navarro P. Colby D. (2012). Esrrb is a direct Nanog target gene that can substitute for Nanog function in pluripotent cells. *Cell Stem Cell* 11 477–490. 10.1016/j.stem.2012.08.002 23040477 PMC3473361

[B9] Goentoro L. Shoval O. Kirschner M. W. Alon U. (2009). The incoherent feedforward loop can provide fold-change detection in gene regulation. *Mol. Cell* 36 894–899. 10.1016/j.molcel.2009.11.018 20005851 PMC2896310

[B10] Hernandez C. Wang Z. Ramazanov B. Tang Y. Mehta S. Dambrot C. (2018). Dppa2/4 facilitate epigenetic remodeling during reprogramming to pluripotency. *Cell Stem Cell* 23 396–411.e8. 10.1016/j.stem.2018.08.001 30146411 PMC6128737

[B11] Hsu S. C. Hung M. C. (2007). Characterization of a novel tripartite nuclear localization sequence in the EGFR family. *J. Biol. Chem.* 282 10432–10440. 10.1074/jbc.M610014200 17283074

[B12] Hutchins A. P. Choo S. H. Mistri T. K. Rahmani M. Woon C. T. Ng C. K. L. (2013). Co-motif discovery identifies an esrrb-Sox2-DNA ternary complex as a mediator of transcriptional differences between mouse embryonic and epiblast stem cells. *Stem Cells* 31 269–281. 10.1002/stem.1279 23169531

[B13] Ivanova N. Dobrin R. Lu R. Kotenko I. Levorse J. DeCoste C. (2006). Dissecting self-renewal in stem cells with RNA interference. *Nature* 442 533–538. 10.1038/nature04915 16767105

[B14] Khulan B. Thompson R. F. Ye K. Fazzari M. J. Suzuki M. Stasiek E. (2006). Comparative isoschizomer profiling of cytosine methylation: the HELP assay. *Genome Res.* 16 1046–1055. 10.1101/gr.5273806 16809668 PMC1524864

[B15] Kim H. J. Osteil P. Humphrey S. J. Cinghu S. Oldfield A. J. Patrick E. (2020). Transcriptional network dynamics during the progression of pluripotency revealed by integrative statistical learning. *Nucleic Acids Res.* 48 1828–1842. 10.1093/nar/gkz1179 31853542 PMC7038952

[B16] Kim J. Chu J. Shen X. Wang J. Orkin S. H. (2008). An extended transcriptional network for pluripotency of embryonic stem cells. *Cell* 132 1049–1061. 10.1016/j.cell.2008.02.039 18358816 PMC3837340

[B17] Kim J. Woo A. J. Chu J. Snow J. W. Fujiwara Y. Kim C. G. (2010). A Myc network accounts for similarities between embryonic stem and cancer cell transcription programs. *Cell* 143 313–324. 10.1016/j.cell.2010.09.010 20946988 PMC3018841

[B18] Kuleshov M. V. Jones M. R. Rouillard A. D. Fernandez N. F. Duan Q. Wang Z. (2016). Enrichr: a comprehensive gene set enrichment analysis web server 2016 update. *Nucleic Acids Res.* 44 W90–W97. 10.1093/nar/gkw377 27141961 PMC4987924

[B19] Lee D. F. Su J. Sevilla A. Gingold J. Schaniel C. Lemischka I. R. (2012). Combining competition assays with genetic complementation strategies to dissect mouse embryonic stem cell self-renewal and pluripotency. *Nat. Protocols* 7 729–748. 10.1038/nprot.2012.018 22441292

[B20] Liu G. David B. T. Trawczynski M. Fessler R. G. (2020). Advances in pluripotent stem cells: history, mechanisms, technologies, and applications. *Stem Cell Rev. Rep.* 16 3–32. 10.1007/s12015-019-09935-x 31760627 PMC6987053

[B21] Loh Y. H. Wu Q. Chew J. L. Vega V. B. Zhang W. Chen X. (2006). The Oct4 and Nanog transcription network regulates pluripotency in mouse embryonic stem cells. *Nat. Genet.* 38 431–440. 10.1038/ng1760 16518401

[B22] Lu R. Markowetz F. Unwin R. D. Leek J. T. Airoldi E. M. MacArthur B. D. (2009). Systems-level dynamic analyses of fate change in murine embryonic stem cells. *Nature* 462 358–362. 10.1038/nature08575 19924215 PMC3199216

[B23] Macarthur B. D. Lachmann A. Lemischka I. R. Ma’ayan A. (2009). GATE: software for the analysis and visualization of high-dimensional time series expression data. *Bioinformatics* 26 143–144. 10.1093/bioinformatics/btp628 19892805 PMC2796822

[B24] Macarthur B. D. Sevilla A. Lenz M. Müller F. J. Schuldt B. M. Schuppert A. A. (2012). Nanog-dependent feedback loops regulate murine embryonic stem cell heterogeneity. *Nat. Cell Biol.* 14 1139–1147. 10.1038/ncb2603 23103910 PMC3507454

[B25] Mangan S. Alon U. (2003). Structure and function of the feed-forward loop network motif. *Proc. Natl. Acad. Sci. U S A.* 100 11980–11985. 10.1073/pnas.2133841100 14530388 PMC218699

[B26] Marson A. Levine S. S. Cole M. F. Frampton G. M. Brambrink T. Johnstone S. (2008). Connecting microRNA genes to the core transcriptional regulatory circuitry of embryonic stem cells. *Cell* 134 521–533. 10.1016/j.cell.2008.07.020 18692474 PMC2586071

[B27] Martello G. Sugimoto T. Diamanti E. Joshi A. Hannah R. Ohtsuka S. (2012). Esrrb is a pivotal target of the Gsk3/Tcf3 axis regulating embryonic stem cell self-renewal. *Cell Stem Cell* 11 491–504. 10.1016/j.stem.2012.06.008 23040478 PMC3465555

[B28] Matoba R. Niwa H. Masui S. Ohtsuka S. Carter M. G. Sharov A. A. (2006). Dissecting Oct3/4-regulated gene networks in embryonic stem cells by expression profiling. *PLoS One* 1:e26. 10.1371/journal.pone.0000026 17183653 PMC1762406

[B29] Milo R. Shen-Orr S. Itzkovitz S. Kashtan N. Chklovskii D. Alon U. (2002). Network motifs: simple building blocks of complex networks. *Science* 298 824–827. 10.1126/science.298.5594.824 12399590

[B30] Nishiyama A. Sharov A. A. Piao Y. Amano M. Amano T. Hoang H. G. (2013). Systematic repression of transcription factors reveals limited patterns of gene expression changes in ES cells. *Sci. Rep.* 3:1390. 10.1038/srep01390 23462645 PMC3589720

[B31] Otasek D. Morris J. H. Bouças J. Pico A. R. Demchak B. (2019). Cytoscape automation: empowering workflow-based network analysis. *Genome Biol.* 20:185. 10.1186/s13059-019-1758-4 31477170 PMC6717989

[B32] Papatsenko D. Darr H. Kulakovskiy I. V. Waghray A. Makeev V. J. Macarthur B. D. (2015). Single-Cell analyses of ESCs reveal alternative pluripotent cell states and molecular mechanisms that control self-renewal. *Stem Cell Rep.* 5 207–220. 10.1016/j.stemcr.2015.07.004 26267829 PMC4618835

[B33] Papatsenko D. Levine M. (2011). The *Drosophila* gap gene network is composed of two parallel toggle switches. *PLoS One* 6:e21145. 10.1371/journal.pone.0021145 21747931 PMC3128594

[B34] Pardo M. Lang B. Yu L. Prosser H. Bradley A. Babu M. M. (2010). An expanded Oct4 interaction network: implications for stem cell biology. *Dev. Dis. Cell Stem Cell* 6 382–395. 10.1016/j.stem.2010.03.004 20362542 PMC2860244

[B35] Shen-Orr S. S. Milo R. Mangan S. Alon U. (2002). Network motifs in the transcriptional regulation network of *Escherichia coli*. *Nat. Genet.* 31 64–68. 10.1038/ng881 11967538

[B36] Unwin R. D. Griffiths J. R. Whetton A. D. (2010). Simultaneous analysis of relative protein expression levels across multiple samples using iTRAQ isobaric tags with 2D nano LC-MS/MS. *Nat. Protocols* 5 1574–1582. 10.1038/nprot.2010.123 21085123

[B37] van den Berg D. L. C. Snoek T. Mullin N. P. Yates A. Bezstarosti K. Demmers J. (2010). An Oct4-Centered protein interaction network in embryonic stem cells. *Cell Stem Cell* 6 369–381. 10.1016/j.stem.2010.02.014 20362541 PMC2860243

[B38] van den Berg D. L. C. Zhang W. Yates A. Engelen E. Takacs K. Bezstarosti K. (2008). Estrogen-Related receptor beta interacts with Oct4 to positively regulate nanog gene expression. *Mol. Cell. Biol.* 28 5986–5995. 10.1128/mcb.00301-08 18662995 PMC2547019

[B39] Verneri P. Vazquez Echegaray C. Oses C. Stortz M. Guberman A. Levi V. (2020). Dynamical reorganization of the pluripotency transcription factors Oct4 and Sox2 during early differentiation of embryonic stem cells. *Sci. Rep.* 10:5195. 10.1038/s41598-020-62235-0 32251342 PMC7089971

[B40] Wang J. Rao S. Chu J. Shen X. Levasseur D. N. Theunissen T. W. (2006). A protein interaction network for pluripotency of embryonic stem cells. *Nature* 444 364–368. 10.1038/nature05284 17093407

[B41] Xu H. Baroukh C. Dannenfelser R. Chen E. Y. Tan C. M. Kou Y. (2013). ESCAPE: database for integrating high-content published data collected from human and mouse embryonic stem cells. *Database* 2013:bat045. 10.1093/database/bat045 23794736 PMC3689438

[B42] Yang J. Gao C. Chai L. Ma Y. (2010). A novel SALL4/OCT4 transcriptional feedback network for pluripotency of embryonic stem cells. *PLoS One* 5:e10766. 10.1371/journal.pone.0010766 20505821 PMC2874005

[B43] You K. T. Park J. Kim V. N. (2015). Role of the small subunit processome in the maintenance of pluripotent stem cells. *Genes Dev.* 29 2004–2009. 10.1101/gad.267112.115 26443847 PMC4604342

[B44] Yu H. B. Kunarso G. Hong F. H. Stanton L. W. (2009). Zfp206, Oct4, and Sox2 are integrated components of a transcriptional regulatory network in embryonic stem cells. *J. Biol. Chem.* 284 31327–31335. 10.1074/jbc.M109.016162 19740739 PMC2781530

[B45] Zhang X. Zhang J. Wang T. Esteban M. A. Pei D. (2008). Esrrb activates Oct4 transcription and sustains self-renewal and pluripotency in embryonic stem cells. *J. Biol. Chem.* 283 35825–35833. 10.1074/jbc.M803481200 18957414

