# Supplementary material for: An Esrrb and Nanog Cell Fate Regulatory Module Controlled by Feed Forward Loop Interactions
Source: Front Cell Dev Biol. 2021 Mar 19;9:630067. doi: 10.3389/fcell.2021.630067 (PMC8017264; doi:10.3389/fcell.2021.630067)
Supplement: Supplementary file 1 [file Presentation_1.pdf]

SUPPLEMENTAL EXPERIMENTAL PROCEDURES

Primer List.

List of oligonucleotides used in this study.

Description	Forward Primer	Reverse Primer	Ref
Esrrb endo	ACCCAGTTCTCAGTGGATGC	AGGAGAAACAGCAGGCAAAA	
Esrrb exo	TCTGGACTCGCCGCCTATGTT	GCGTTAAGCATGTACTCGCATTTGAT	
Intergenic_Chr8	AAGGGGCCTCTGCTTAAAAA	AGAGCTCCATGGCAGGTAGA	Boyer et al. 2006.
Intergenic_Chr6	CCCCTTTCTGAAGCACTCTG	TAAGGCGTCATTTCCCAAAG	Boyer et al. 2006.
pSox2	CCTAGGAAAAGGCTGGGAAC	GTGGTGTGCCATTGTTTCTG	Boyer et al. 2006.
pPou5f1	TGGGCTGAAATACTGGGTTC	TTGAATGTTCGTGTGCCAAT	Boyer et al. 2006.
pNanog	TTGGGACCAGCTAGAGCAAT	CCAGGCTTGTCTACCACCAT	Boyer et al. 2006.
pSox17	TGTCCTTTAACAGGCCTTGG	GCAGCGGTATCACACTCAAA	Boyer et al. 2006.
pOlig1	CCATCTGTTGCAAAATCGTG	CTCGAGCTCTATTGCCATCC	Boyer et al. 2006.
pOlig2	GCCTGACGCTACAGTGACAA	GGCTAATTCCGCTCAATGAA	Boyer et al. 2006.
pOlig3	CTGAATGATGATGGCACGTC	CTGTTTCCTGCACTGCACAT	Boyer et al. 2006.
pZfp42Fwd (–283 to –117)	TGCATCCTCTGCTTGTGTAA	CAGAGCTGTCCCCTTGTCT	van der Berg.D 2008
pRest Fwd (–3216 to –3071)	CTCCCCTGGACAATAGCTTC	CGTCCTTCATTTCCTCAGTG	van der Berg.D 2008
pDppa3 Fwd (–1770 to –1550)	GATCCAGCTGGTCTGAGCTA	GTGCAGGGATCATAGGAGTG	van der Berg.D 2008
pLefty1 Fwd (–1264 to –1060)	AAGCTGCAGACTTCATTCCA	CGGGGGATAGATGAAGAAAC	van der Berg.D 2008
pAmilase	CTCCTTGTACGGGTTGGT	AATGATGTGCACAGCTGAA	van der Berg.D 2008
Rnf125	TGACATGAGGGCGCACATAAG	TGGTGTCGCCAAGTTCTAGC	

Zscan10	GCGGAACCAGTCCCTGATG	CGTGGCCCCATATCTTCCTG	
Dppa2	TCAACGAGAACCAATCTGAGGA	GCGTAGCGTAGTCTGTGTTTG	
Krr1	CTCAGCCGCAGAAACAGAAG	ATTTCGGGAACAAGGTTGCGA	
Esrrb	CTAGTTGCGGCTCCTTCATC	TGGCGTTAAGCATGTACTCG	
Nanog	CCAGTCCCAAACAAAAGCTC	ATCTGCTGGAGGCTGAGGTA	
Pou5f1	GGATGGCATACTGTGGACCT	GTTGGTTCCACCTTCTCCAA	
Sox2	CACAACTCGGAGATCAGCAA	CTCCGGGAAGCGTGTACTTA	
Fgfr2	CACCGAGAAGATGGAGAAGC	GCGATGCTCCTGCTTAAACT	
Gli2	GCCCTGGAGAGTCACCCTT	TGCACAGACCGGAGGTAGT	
Mllt6	ATGAAGGAGATGGTAGGAGGC	CCTGGACGATGCCATAGCAA	
Nefl	CCGTACTTTTCGACCTCCTACA	CTTGTGTGCGGATAGACTTGAG	
Trim25	ATGGCTCAGGTAACAAGGGAG	GGGAGCAACAGGGGTTTTCTT	
Spred2	GAAGAAACACACCCGGACGAT	CTCCTTCCTGTGGGAACCAT	
Sumo3	GGCTCGGTGGTACAGTTCAAG	CCGGAATCGAATCTGCCTCAT	

Real-time quantitative PCR

Cells were trypsinized and collected at specific time points. Total RNA was extracted using Trizol Reagent (Invitrogen), column-purified with RNeasy kit (Qiagen) and treated with RNase-free DNase (Qiagen). Total RNA (1 µg) was reverse transcribed using a high-capacity reverse transcription kit (Applied Biosystems). All quantitative PCR analyses were performed using the Fast SYBR Green Master Mix (Applied Biosystems) following the manufacturer’s protocols on the Light Cycler 480 Real-Time PCR System (Roche). All measurements were performed in technical triplicates.

Antibodies

The following commercially available antibodies were used at the indicated concentrations for western blot: α–β-actin (Sigma, catalogue number A5441, clone AC-15, RRID: AB_476744 1:1,000), Esrrb (R&D Systems, PP-H6707, RRID: AB_1961870, 1:1000), H3 (Abcam, ab1791, RRID: AB_302613, 1:1000), H3K4me3 (Abcam, ab8580, RRID: AB_306649, 1:1000), H3K27me3 (Upstate, 07-449, RRID: AB_310624, 1:1000), GFP (Cell Signaling, 2555, RRID: AB_10692764

1:1000), α-Tubulin (Sigma, T5168, RRID: AB_477579, 1:1000), Calnexin (Sigma, C4731, RRID: AB_476845, 1:1000), Dnmt3a (Abgent, AP1034a, RRID: AB_352513, 1:500), Mov10 (Protein Tech Group, 10370-1-AP, RRID: AB_2297897, 1:1000), Klf5 (Abcam, ab24331, RRID: AB_775925, 1:1000), CREB1(Santa Cruz, sc-58, RRID: AB_631314, 1:500).

Microarray data analysis

Significant genes for each time series were found using one-way analysis of variance with a Bonferroni-corrected *P* value of 0.05 and a total fold change greater than 2. All subsequent clustering and dimensionality reduction analyses were performed in R and Matlab, using the Bioinformatics and Statistics Toolboxes. Data were row standardized to avoid bias towards highly expressed genes and hierarchical clustering was performed on standard scores. Hierarchical clustering was performed using the Euclidean distance metric and the average linkage function.

Processing the proteomic data

We used ProQUANT and ProGROUP software (Applied Biosystems) to analyze the mass spectrometric data, giving confidence values for the relative quantification analysis. Further proteomic analyses included only proteins that were identified with more than 95% confidence and where the error factor of the measurement was less than 2. From the 1790 detected proteins, the analysis considered only that mapped to our non-redundant mRNA data set (**Table S1**).

Chromatin Immunoprecipitation

Chromatin Immunoprecipitations (ChIP) were performed as described (Boyer et al., 2005). Images acquired from the Solexa sequencer were processed through the bundled Solexa image extraction pipeline and aligned to the Mouse July 2007 assembly (NCBI37/mm9) using ELAND software. Complete sequence data are available at the NCBI GEO (Gene Expression Omnibus) database (Edgar et al., 2002; Barrett et al., 2011) under the accession designation number: GSE31842. Only uniquely mapped coordinates were used for further analyses. Uniquely aligned 36 bp sequences were extended to 250 bp in the 3’ direction and allocated into 20 bp bins. We used a previously published algorithm (Zhang et al., 2008), Model-based Analysis of ChIP-Seq (MACS), for peak-finding.

Binding regions were identified based on: (1) 250 bp bandwidth, (2) >10-fold and <30–fold change between total tag counts from each ChIP library vs. the negative/input ChIP library and (3) a p-value threshold of 10-5. Esrrb bound genomic DNA was enriched from whole cell lysates using anti-Esrrb antibody (R&D; PP-H6707-00, Clone H6707, Lot# A-1, RRID: AB_1961870) and compared to the starting whole cell extracts. H3K4me3 and H3K27me3 modified nucleosomes were enriched from whole cell lysates at the day 0, 1, 3 and 5 time points using an epitope-specific rabbit polyclonal antibody for H3K4me3 (Abcam; ab8580, Lot #1016899, RRID: AB_306649) and H3K27me3 (Upstate: 07-449; Lot# DAM1387952, RRID: AB_310624)(Santos-Rosa et al., 2002; Guenther et al., 2007) and compared to the starting whole cell extract corresponding to the each specific day.

Array-based methylation analysis using HELP

The HELP assay was performed as previously described (Khulan et al., 2006) with the following modifications. One microgram of high molecular weight DNA was digested overnight with HpaII or MspI (NEB, Ipswich, MA). DNA fragments were purified using phenol/chloroform, resuspended in 10 mM Tris-HCl pH 8.0, and ligated in an overnight reaction using the HpaII aptamer and T4 DNA ligase. Ligation-mediated PCR was performed as previously described (Khulan et al., 2006) and products were labelled and hybridized onto a Roche NimbleGen, Inc Arrays (Madison, WI) custom array designed by Dr. Ari Melnick's laboratory (Weill Cornell medical College, NY).

Array-based methylation using HELP data analysis

Data pre-processing and quality control was performed as previously described (Khulan et al., 2006) using R software 2.9.0 (2008) and BioConductor (Gentleman et al., 2004). Intra-array normalization was followed by the determination of background noise for each channel (2.5 median absolute deviations from the median of the random probes log2 signal for that channel). Each channel was centered by subtracting this noise threshold from its log2-transformed signal intensities.The HpaII/MspI (unmethylated/reference) ratio was then determined for each probe set on the array.

The position of the centre of each probe on the array was compared with the transcription start site (TSS) of known Refseq retrieved from UCSC mouse genome annotations Mouse July 2007 (NCBI37/mm9) assembly. Differentially methylated probe sets between the day 0 and days 1, 3 and 5 were determined by the one-way ANOVA test (Ayroles and Gibson, 2006). The significance threshold was set to ANOVA p-value<0.05, and concurrently FDR was determined using the Benjamini and Hochberg method (Benjamini and Hochberg, 1995). In addition, our threshold for differential methylation for each gene between the means of day 0 and days 1, 3 and 5 was greater than 20% (i.e. ratio of the mean methylation in the two groups [Δ log (HpaII/MspI)] >= 1.5). **Table S4** contains the complete lists of differentially methylated genes for Esrrb and Nanog knockdown time series within 1Kb from the TSS.

Data integration analysis of all the regulatory layers

Merging different regulatory layers: Datasets for the six regulatory layers (mRNA transcriptome, DNA methylome, nuclear proteome, (histone H3 trimethylated at lysine 4 (H3K4me3), histone H3 trimethyl Lys27 (H3K27me3), and Esrrb localization) were merged by matching each data type to 34,662 non-redundant gene names, available at the MGI (Blake et al., 2011) open web resource:

(ftp://ftp.informatics.jax.org/pub/reports/MRK_Synonym.rpt). Genes, dismissed (reassigned) in the most recent annotation of Mouse July 2007 (NCBI37/mm9) assembly and gene name synonyms as well as RefSeq (Pruitt et al., 2000) were taken into account, in order to minimize losses at the dataset integration stage. Using this procedure, it was possible to match 95%-99% of data points for each regulatory layer. Merging has been done after analysis of variance ANOVA (Ayroles and Gibson, 2006) to score the significance of the correction (as described in detail see below).

Normalization of time series data from the multiple layers: Time-series data for all layers were normalized to day 0 using Z-score normalization:

$$Z_{i,j} = \frac{x_{i,j} - x_{i,0}}{\sigma_i} \quad (1)$$

In this formula, σ is the standard deviation in the time series (row) *i* and $x_{i,0}$ is the value for the day (column) 0. The main advantage of this procedure is that it produces the same (comparable) range of values for each profile in each regulatory layer. Normalization was done after the layers were merged together.

Processing the mRNA transcriptome expression and DNA methylome layers: Time-series of gene expression data and DNA methylation data, obtained from the Esrrb knockdown time-course, were first subjected to standard ANOVA test (Ayroles and Gibson, 2006). The ANOVA evaluates the consistency of expression values for each probe from one biological replica to another. In order to correct for multiple hypothesis testing, false discovery rates (FDR) (Benjamini and Hochberg, 1995) were computed based on the ANOVA p-values as follows:

$$P_i^{adj} = \min\left(P_{i-1}^{adj}, \frac{N}{k} P_i \right); P_1^{adj} = P_1 \quad (2)$$

P in eq.2 is the ANOVA p-value, P^{adj} is the adjusted p-value for multiple hypothesis testing, *N* is the total number of samples (number of probes in the microarray) and *k* is the rank of the ith probe, with respect to the ANOVA p-values (lowest ANOVA p-value is the 1st rank, highest ANOVA p-value is the Nth rank). In general, the FDR test is “softer” than the more common Bonferroni correction (Sture Holm, 1979).

Analysis of multiple regulatory layers suffers from missing data; therefore, the threshold for filtering of expression and methylome data (1Kb from TSS only) was set to a relatively low level to include of as many data points as possible. For the methylome we set the threshold at 15% of FDR (15% of false positive can be tolerated). After filtering the data based on this FDR (before merging with other layers), genes with multiple profiles (ie. probes from the array with different methylation states in the CpG islands corresponding to the same gene) were assigned a single profile, choosing the probe with the best p-value. The described procedure produced 11,324 significantly changing genes for the expression data and 4495 significantly changed genes from the methylome data.

Processing the proteome layer: The proteome layer measured nuclear protein levels because cell fate determination is largely controlled in the nucleus. Therefore, the proteome dataset only measured a small subset of gene-products as compared with the other layers. Nonetheless, we identified 1790 proteins after merging IDs with MGI, as described above (**Table S1**).

Processing the Esrrb ChIP-seq and histone modification layers: MACS model-based analysis for ChIP-seq scores for the H3K4me3, H3K27me3 and Esrrb datasets were selected for 2Kb upstream regions only; redundant profiles for each gene were filtered and single profiles (or data points for Chip-seq data), giving the best MACS score were retained. Consequently, the data were merged relative to the non-redundant set of 34,662 genes from MGI (see above). Based on cross-layer comparisons (ChIP-

seq versus expression), it was determined that 600 Esrrb target genes are statistically differentially expressed based on the ANOVA (Ayroles and Gibson, 2006) with the p-value 0.078 and FDR 15%.

Chromatin modification regulatory layers (H3K4me3 and H3K27me3 marks) were also filtered for redundant profiles; 2Kb from the TSS was selected for the analyses and the datasets were normalized as described above.

Meta-clustering multidimensional time-series and data visualization using Cytoscape

1563 genes and gene-products with changes in any three or more out of the six regulatory layers analyzed were selected for meta-clustering. Co-expression distance matrix has been constructed based on probabilities calculated for the corresponding pairwise correlation values *r* given the number of available for the comparison data points *N*:

$$d^{A-B} = p(r^{A-B}, N) \qquad (3)$$

If the data for a given dimension was missing, the *N* takes this into account, thus making all data comparable even in the absence of detected changes in any given dimension for any given pair of genes. On the example below, comparison between the genes A and B is only available for two dimensions, mRNA and H3K4, but not for the protein dimension. In this case, the correlation is calculated only for 8 data points (*N*=8) marked in red.

	mRNA				protein				H3K4				...
	Day0	Day1	Day3	Day5	Day0	Day1	Day3	Day5	Day0	Day1	Day3	Day5	...
Gene A	data	data	data	data	**NA**	**NA**	**NA**	**NA**	data	data	data	data	...
Gene B	data	data	data	data	Data	data	data	data	data	data	data	data	...

Network layout and visualization was done using the publicly available Cytoscape (Otasek et al., 2019). http://www.cytoscape.org/. The two-dimension clustering was constructed in the form of co-expression gene networks using a force-directed Cytoscape layout. First, a Pearson similarity matrix was generated for the 1563 genes using all six regulatory layers for every gene. Second, a similarity cut-off value was established in order to identify the most significant correlations between the genes. The cut-off value was selected based on the connectivity of the resulting co-expression gene networks: high cut-off values produced many unconnected networks with low numbers of edges and low cut-off values produced high connectivity networks containing very large numbers of network edges. In this study the lowest possible similarity cut-off value was found, where all major network parts are connected. Both highly negative and highly positive correlations were considered ($r < -0.8$; $r > 0.8$). Based on this cut-off definition, the co-expression gene network in *.gml format (graph mark-up language) was constructed and imported into Cytoscape. Force-directed Cytoscape layout repeatedly produced the same topology even with slight variations in layout parameters. **Table S1** contains all the data integrated and normalized for all the regulatory layers analyzed.

The role of Esrrb in the transcriptional hierarchy of pluripotency gene network. Integration of *in vivo* binding (ChIP) data and expression data

In order to establish the position of Esrrb in the transcriptional hierarchy we collected and integrated two types of data for the three core pluripotency factors (Oct4, Nanog, Sox2) and Esrrb. The data included (*i*) the results of knockdown or knockout expression microarray studies from two to three independent sources and (*ii*) the results of *in vivo* chromatin immunoprecipitation studies from up to three independent sources.

Three knockdown expression datasets were found and taken into consideration for Esrrb (Ivanova et al., 2006; Feng et al., 2009) (plus one Esrrb dataset produced in this study), Nanog (Ivanova et al., 2006; Loh et al., 2006; Macarthur et al., 2012) Oct4 (Ivanova et al., 2006; Loh et al., 2006) and Sox2 (Ivanova et al., 2006). Consequently, all analyzed expression data sets were processed in a similar way: raw data was converted to log-space. Significance of gene expression changes at the Day 1 versus Day 0 has been found from 2-tailed, 2-sample equal variance Student's t-test. Such analysis reveals genes quickly responding to concentration changes of the upstream regulator; so, the pool of the identified genes should contain many of the direct targets for the analyzed transcriptional regulator.

Direction of the immediate gene expression changes (sign, upregulation or downregulation) has been monitored in order to find whether the upstream regulator plays role as an activator or as a repressor for the downstream genes. Genes producing conflicting results with respect to different datasets were removed from consideration.

A score S_e reflecting the level of confidence has been assigned to each gene based on the number of knockdown expression studies supporting the gene's immediate response. If no significant changes were observed in two out of the three studies, we assigned $S_e = 0$, for two supporting studies we assigned $S_e = 1$ and if supported by all three studies $S_e = 2$. Many transcription factors produced the highest expression score ($S_e = 2$): Oct4 - 51 genes, Nanog - 14 genes, Esrrb - 18. Only 2 suitable datasets were found for Sox2, correspondingly the highest score in the case of Sox2 was $S_e = 1$ (55 transcription factors).

ChIP data has been collected for each of the considered four pluripotency factors (Oct4, Nanog, Sox2, Esrrb)(Chen et al., 2005; Kim et al., 2008; Marson et al., 2008), including ChIP data for Esrrb produced in the current study. In the case of Oct4, Nanog and Sox2 ChIP data we compared 3 datasets and assigned a binding score Sb = 1 to genes, reported as targets in two or three out of the three considered datasets and Sb = 0 to all other genes.

Based on the described above expression (S_e) and the chip (S_b) scores, a combined evidence score S was calculated based on the following rule:

$$S = S_e + S_b \text{ for } Se > 0 \quad (4)$$

$$S = 0 \text{ for } Se = 0$$

Table S5 shows the corresponding scores for all four considered core pluripotency factors. A signed and directed gene network, combining data from **Table S5** is shown in **Figure 5.** According to the rank analysis shown in the **Table S5**, Sox2 and Nanog occupy ranks #12 and #23 among transcription factors, Esrrb targets. Given 1578 mouse transcription factors considered in total, this translates to 0.8% and 1.5% top percentile or q=0.0076 and q=0.0146 correspondingly. Even more strikingly, Esrrb occupies the rank #2 among the Nanog targets (#3 is Sall4) and the same rank #2 among the Sox2 targets (rank #1 is Klf4). Among the Oct4 targets Esrrb occupies rank #25, higher than Sox2 (#64) and Nanog (#108).

The above analysis suggests that Esrrb is tightly linked to the other core pluripotency factors and it occupies the central position in the transcriptional hierarchy.

DATA AVAILABILITY STATEMENT

All microarray data and ChIP-seq data are deposited at the Gene Expression Omnibus (GEO) database repository under accession number GSE-31842. https://www.ncbi.nlm.nih.gov/geo/query/acc.cgi?acc=GSE31842. This link gives access to all data sets uploaded in this database repository organized in four different subseries GSE31640, GSE31791 and GSE33191.

SUPPLEMENTAL REFERENCES

Ayroles, J. F., and Gibson, G. (2006). [11] Analysis of Variance of Microarray Data. *Methods Enzymol.* 411, 214–233. doi:10.1016/S0076-6879(06)11011-3.

Barrett, T., Troup, D. B., Wilhite, S. E., Ledoux, P., Evangelista, C., Kim, I. F., et al. (2011). NCBI GEO: Archive for functional genomics data sets-10 years on. *Nucleic Acids Res.* 39. doi:10.1093/nar/gkq1184.

Benjamini, Y., and Hochberg, Y. (1995). Controlling the False Discovery Rate: A Practical and Powerful Approach to Multiple Testing. *J. R. Stat. Soc. Ser. B* 57, 289–300. doi:10.1111/j.2517-6161.1995.tb02031.x.

Blake, J. A., Bult, C. J., Kadin, J. A., Richardson, J. E., and Eppig, J. T. (2011). The mouse genome database (MGD): Premier model organism resource for mammalian genomics and genetics. *Nucleic Acids Res.* 39. doi:10.1093/nar/gkq1008.

Boyer, L. A., Tong, I. L., Cole, M. F., Johnstone, S. E., Levine, S. S., Zucker, J. P., et al. (2005). Core transcriptional regulatory circuitry in human embryonic stem cells. *Cell* 122, 947–956. doi:10.1016/j.cell.2005.08.020.

Chen, C., Ridzon, D. A., Broomer, A. J., Zhou, Z., Lee, D. H., Nguyen, J. T., et al. (2005). Real-time quantification of microRNAs by stem-loop RT-PCR. *Nucleic Acids Res.* 33. doi:10.1093/nar/gni178.

Edgar, R., Domrachev, M., and Lash, A. E. (2002). Gene Expression Omnibus: NCBI gene expression and hybridization array data repository. *Nucleic Acids Res.* 30, 207–210. doi:10.1093/nar/30.1.207.

Feng, B., Jiang, J., Kraus, P., Ng, J. H., Heng, J. C. D., Chan, Y. S., et al. (2009). Reprogramming of fibroblasts into induced pluripotent stem cells with orphan nuclear receptor Esrrb. *Nat. Cell Biol.* 11, 197–203. doi:10.1038/ncb1827.

Gentleman, R. C., Carey, V. J., Bates, D. M., Bolstad, B., Dettling, M., Dudoit, S., et al. (2004). Bioconductor: open software development for computational biology and bioinformatics. *Genome Biol.* 5. doi:10.1186/gb-2004-5-10-r80.

Guenther, M. G., Levine, S. S., Boyer, L. A., Jaenisch, R., and Young, R. A. (2007). A Chromatin Landmark and Transcription Initiation at Most Promoters in Human Cells. *Cell* 130, 77–88. doi:10.1016/j.cell.2007.05.042.

Ivanova, N., Dobrin, R., Lu, R., Kotenko, I., Levorse, J., DeCoste, C., et al. (2006). Dissecting self-renewal in stem cells with RNA interference. *Nature* 442, 533–538. doi:10.1038/nature04915.

Khulan, B., Thompson, R. F., Ye, K., Fazzari, M. J., Suzuki, M., Stasiek, E., et al. (2006). Comparative isoschizomer profiling of cytosine methylation: The HELP assay. *Genome Res.* 16, 1046–1055. doi:10.1101/gr.5273806.

Kim, J., Chu, J., Shen, X., Wang, J., and Orkin, S. H. (2008). An Extended Transcriptional Network for Pluripotency of Embryonic Stem Cells. *Cell* 132, 1049–1061. doi:10.1016/j.cell.2008.02.039.

Kuleshov, M. V., Jones, M. R., Rouillard, A. D., Fernandez, N. F., Duan, Q., Wang, Z., et al. (2016). Enrichr: a comprehensive gene set enrichment analysis web server 2016 update. *Nucleic Acids Res.* 44, W90–W97. doi:10.1093/nar/gkw377.

Loh, Y. H., Wu, Q., Chew, J. L., Vega, V. B., Zhang, W., Chen, X., et al. (2006). The Oct4 and Nanog transcription network regulates pluripotency in mouse embryonic stem cells. *Nat. Genet.* 38, 431–440. doi:10.1038/ng1760.

Macarthur, B. D., Sevilla, A., Lenz, M., Müller, F. J., Schuldt, B. M., Schuppert, A. A., et al. (2012). Nanog-dependent feedback loops regulate murine embryonic stem cell heterogeneity. *Nat. Cell Biol.* 14, 1139–1147. doi:10.1038/ncb2603.

Marson, A., Levine, S. S., Cole, M. F., Frampton, G. M., Brambrink, T., Johnstone, S., et al. (2008). Connecting microRNA Genes to the Core Transcriptional Regulatory Circuitry of Embryonic Stem Cells. *Cell* 134, 521–533. doi:10.1016/j.cell.2008.07.020.

Otasek, D., Morris, J. H., Bouças, J., Pico, A. R., and Demchak, B. (2019). Cytoscape Automation: Empowering workflow-based network analysis. *Genome Biol.* 20, 185. doi:10.1186/s13059-019-1758-4.

Pruitt, K. D., Katz, K. S., Sicotte, H., and Maglott, D. R. (2000). Introducing RefSeq and LocusLink: Curated human genome resources at the NCBI. *Trends Genet.* 16, 44–47. doi:10.1016/S0168-9525(99)01882-X.

Santos-Rosa, H., Schneider, R., Bannister, A. J., Sherriff, J., Bernstein, B. E., Emre, N. C. T., et al. (2002). Active genes are tri-methylated at K4 of histone H3. *Nature* 419, 407–411. doi:10.1038/nature01080.

Sture Holm (1979). A Simple Sequentially Rejective Multiple Test Procedure. *Scand. J. Stat.* 6, 65–70. Available at: https://scholar.google.es/scholar?hl=en&as_sdt=0%2C5&as_vis=1&q=A+simple+sequentially+rejective+multiple+test+procedure.+Scandinavian+Journal+of+Statistics+6%2C+65–70.&btnG= [Accessed October 23, 2020].

Zhang, X., Zhang, J., Wang, T., Esteban, M. A., and Pei, D. (2008). Esrrb activates Oct4 transcription and sustains self-renewal and pluripotency in embryonic stem cells. *J. Biol. Chem.* 283, 35825–35833. doi:10.1074/jbc.M803481200.

SUPPLEMENTARY FIGURE LEGENDS

Figure S1. Esrrb rescue clone and experimental time course controls for different regulatory layers. In relation to Figure 1.

(A) Esrrb expression levels after Dox withdrawal. Endogenous levels of Esrrb mRNA are undetectable due to the constitutive shRNA (see Fig. 1A). Quantitative PCR data confirm expression of the exogenous form in presence of Dox (mean ± SD for 3 replicates (n=3)). Lower panel, Western blot confirms downregulation of Esrrb and GFP in the absence of Dox. (B) qPCR analysis of early lineage differentiation markers. (C) Differentiation of mESCs shown by gradual changes in morphology and loss of alkaline phosphatase (AP) activity in cells maintained for 5 days without Dox. In presence of Dox cells maintain an undifferentiated phenotype. (D) Flow cytometry analyses of SSEA1 cell surface marker levels upon depletion of Esrrb. (E) Specificity of the anti-Esrrb antibody used for ChIP. Enrichment of Esrrb-bound chromatin was confirmed by Western blots using IgG as a non-specific control. The arrow indicates Esrrb. (F) Distribution of Esrrb ChIP-seq tags relative to the TSS. Tag counts were normalized to the total number of tags in each sequencing reaction. (G) Subcellular localization controls for iTRAQ samples. Equal amounts of total protein from cytosolic and nuclear fractions were analyzed by Western blots using anti-Tubulin (cytosolic marker), anti-Calnexin (endoplasmic reticulum marker) and anti-histone-H3 (nuclear marker). Western blot confirmation of Esrrb depletion and nuclear localization over the depletion time-course. (H) Hierarchical clustering of the significant $\log_2$ fold protein level changes during the time-course. (I) Protein levels measured by iTRAQ correlate with Western blot results for Dnmt3a, Mov10, Klf5 and Creb1.

Figure S2.Visualization of dynamic changes across the analysed data dimensions. In relation to Fig. 2.

The dynamic changes are shown by colours in the context of the clusters shown in the Fig. 2. The colour scale changes from red (maximal expression) to green (absence of expression), see also the

colour bar in the left top corner. (Panels A-D) Changes in the mRNA levels, (E-H) Changes in the protein levels, (I-L) Changes in the promoter methylation, (M-P) Changes in the level of H3K4me3 methylation, (Q-T) Changes in the level of H3K27me3 methylation. The dynamics has been also represented in Movie S1.

Figure S3. Standard deviations in promoter methylation data. DNA methylome dynamics after Esrrb or Nanog depletion. In relation to Fig. 2.

(A) The figure shows errors in measurement of promoter methylation for genes shown in the Fig. 2J. The errors were calculated based on biological replicas and are represented as standard deviations interpolated to the entire time course of differentiation. (B) Hierarchical clustering of the set of genes with significant changes in promoter methylation after depletion of Esrrb or Nanog. GO categories based on Enrichr (Kuleshov et al., 2016) are depicted for the groups of genes whose methylation profiles follow the same trend or an opposite trend in the Esrrb versus Nanog time course.

Figure S4. Co-expression clustering of mRNA and protein time series data.

(A) The figure shows co-expression clustering of data containing genes significantly changing their mRNA and their protein levels. Cluster #1 shows genes upregulated upon Esrrb removal in both analysed dimensions. This cluster contains many differentiation markers, such as Gata6. Cluster #5 shows genes downregulated upon Esrrb, this cluster contains most pluripotency genes (labelled in red). Cluster #4 contains genes, where protein expression is delayed relatively to mRNA expression, Clusters #3 and #2 contain genes, where various significant discordances between the mRNA and the protein levels are observed. The dynamics has been also represented in Movie S2.
